# Climacteric Menorrhagia

**Published:** 1867-02

**Authors:** B. F. Barker

**Affiliations:** Bellevue Medical College


*Climacteric Menorrhagia.*—Extract from a Lecture Delivered by Prof. B. F. Barker, at Bellevue Medical College, Dec. 11, 1866.

In the early part of my practice, and for many years, I met with a class of patients, from forty-three to forty-five years of age, who suffered from a constant flow of the menses, about two weeks in every four, a constant draft upon the genital organs, which was accompanied with great disturbance of the nervous system, and of the general health. I examined for polypi, fibrous tumors, and cancer, with the greatest care. I found it to be a peculiar menorrhagia, occurring especially at that period of life when the functions of ovulation and menstruation were about to cease. I could find no organic disease upon which it appeared to depend. I consulted all the works to which I could gain access, both in our own and the French languages, with especial reference to this point; but found nothing satisfactory to aid me in its treatment. I find that, even at this time, none of our most recent authors, as Scanzoni, Bennett, McClintock, West, and Huett, or of the French authors, make any especial allusion to this peculiar type of menorrhagia, which I call the *climacteric menorrhagia.*

I feel assured, that in actual practice, you will frequently meet with these cases, and will find the directions of authors vague and unsatisfactory, and the proper management of the affection very perplexing.

Climacteric menorrhagia occurs in the plethoric and the anæmic, and I believe it is no more liable to occur in the one habit of the system than in the other. It continues for a longer or shorter time, according to the improper or proper treatment of it. It is not an affection susceptible of arrest or cure by any constitutional measures. The ordinary styptics, hæmostatics, and astringents have very little influence in controlling it. Churchill, in speaking of the treatment of this form of menorrhagia, recommends ergot as especially valuable in that class of cases where it is associated with an enlarged or hypertrophied uterus. Other authors recommend a great variety of styptics; but none of them speak with any very great assurance regarding the effects of their remedies.

When this menorrhagia accurs in those of full habit, and continues, as it often does, two, three, or more years, the patient is reduced from a plethoric to an anæmic condition. It frequently renders the condition of the general system such that it is incapable of bearing with the ordinary normal resistance, the shock of the little accidental injuries which may arise.

It sometimes appears in the form of violent exhausting hemorrhage, to an extent almost equal to that which may occur in the puerperal state; this to be followed by an arrest of the menstrual function for two or three months, or longer, when a second hemorrhage takes place, and so on. The system is thus reduced to a condition of anæmia, from which it arises only to be again brought down by this rupture of, and excessive hemorrhage from, the capillary vessels of the uterus. In other cases, the drain is less profuse and exhausting, and occurs much more frequently. These are no fancy pictures, but what I frequently meet with in actual practice.

A few years ago, I came to the conclusion that this form of menorrhagia was due to no peculiar condition of the general system, was caused or accompanied by no organic lesion, and was uninfluenced by constitutional treatment. I then began to reflect, that the internal surface of the uterus, during the whole period of what is termed menstrual life, is subjected to a constant process of formation and development of the mucous membrane and its follicles; the decidual membrane, its exfoliation and reproduction, occurring to a certain extent at each menstrual period, and at each period of gestation and parturition, to a full extent; that this process might be interrupted, and that it was possible and probable that these forms of menorrhagia were due to the condition of the internal surface of the uterus. I now believe that this form of menorrhagia is due to an imperfect cicatrization of the lining membrane of the uterus, following the exfoliation which occurs at each menstrual period, and associated with increased vascularity of this membrane. In other words, I believe it is caused by a lesion of the internal surface. I commenced treatment in accordance with this belief, and its success convinces me that the theory is sound.

I have, within a few years past, conversed with certainly the most eminent uterine pathologists now living in different parts of the world, and when the proposition has been fully stated to them, it has been accepted; and the treatment I propose, is now being practiced in Edinburgh, London, and Paris, as well as by many in our own country.

Tepid water injected into the cavity of the uterus, in its normal undeveloped state, even in quantities of ten or fifteen drops, causes the most intense uterine colic. It is infinitely more tolerant of solid substances than of fluids, even the most bland. The presence of fluid seems to excite uterine contraction, and the uterine muscular tissue not being developed, these attempts at contraction produce excessive pain. But in these cases of profuse uterine hemorrhage, where the cavity is enlarged and its capacity increased, half an ounce or more of fluid may be thrown in and retained by the then tolerant uterus.

Since Dr. Squibb, to whom the profession is deeply indebted, introduced the solution of the persulphate of iron, I have used this exclusively in the cases of sudden and excessive hemorrhage, where there is consequently more or less blood accumulating in the uterus. In these cases I use an india-rubber syringe of the ordinary form for uterine injections. If the injection is thrown in with any force, it will be thrown out at once. The object is to have it retained in the cavity of the uterus, to exert its styptic influence, which is to form a firm coagulum, thus blocking up the open mouths of the vessels, and also to astringe the coats of the vessels themselves. So I introduce the syringe, and, with great gentleness, inject from twenty to forty drops of the persulphate of iron. I have never, in a single instance, made use of this remedy without its being immediately followed by a complete and entire cessation of hemorrhage, and no recurrence for some days afterward. In these forms of dangerous, profuse, and sudden hemorrhage, this treatment is infinitely more successful than any other measure.

The second form of menorrhagia, is that which occurs at the climacteric period, which is much less profuse, but continues from two to three weeks, an exhaustive drain upon the system. In this form, my object is to carry into the cavity of the uterus some substance which will produce rapid cicatrization of its lining surface. The agent which I have been using exclusively for this purpose, for five or six years, is a solution of sulphate of zinc in glycerine. The combination is:—

℞. Zinci Sulphatis,------------------------ ℥j.

Glycerin,--------------------------- f.℥ij.

I find that a drachm of glycerine will dissolve an ounce of the sulphate of zinc; but in the combination I have given you, the proportions are such as to give it the proper consistence.

The instrument I use for this purpose is a hollow tube perforated in every direction at its extremity, the whole shaped like an ordinary female catheter, in which is fitted a piston. The instrument is filled sufficiently by partially withdrawing the piston, and dipping the perforated extremity into the ointment, which is then injected into the cavity of the uterus, by the use of the piston. In regard to this treatment, I could give you a great number of cases, in which the patients, in a state of extreme exhaustion, have been subjected to a great variety of constitutional and local treatment, but without effect, until by means of this simple treatment, the drain upon the system has been arrested. In these cases of menorrhagia, I have not, in a single one, had to apply this method more than twice.—*Phila. Med. & Surg. Reporter.*

